# The Beauty of Asymmetric Membranes: Reconstitution of the Outer Membrane of Gram-Negative Bacteria

**DOI:** 10.3389/fcell.2020.00586

**Published:** 2020-07-14

**Authors:** Laura Paulowski, Annemarie Donoghue, Christian Nehls, Sabrina Groth, Max Koistinen, Sven O. Hagge, Arne Böhling, Mathias Winterhalter, Thomas Gutsmann

**Affiliations:** ^1^Division of Biophysics, Priority Research Area Infection, Research Center Borstel Leibniz Lung Center, Borstel, Germany; ^2^Division of Diagnostic Mycobacteriology, Priority Research Area Infection, National Reference Center for Mycobacteria, Research Center Borstel Leibniz Lung Center, Borstel, Germany; ^3^Department of Life Sciences & Chemistry, Jacobs University Bremen, Bremen, Germany

**Keywords:** phase transfer, membranes, asymmetry, coupling, lipid flip-flop

## Abstract

The architecture of the lipid matrix of the outer membrane of Gram-negative bacteria is extremely asymmetric: Whereas the inner leaflet is composed of a phospholipid mixture, the outer leaflet is built up by glycolipids. For most Gram-negative species, these glycolipids are lipopolysaccharides (LPS), for a few species, however, glycosphingolipids. We demonstrate experimental approaches for the reconstitution of these asymmetric membranes as (i) solid supported membranes prepared by the Langmuir-Blodgett technique, (ii) planar lipid bilayers prepared by the Montal-Mueller technique, and (iii) giant unilamellar vesicles (GUVs) prepared by the phase transfer method. The asymmetric GUVs (aGUVs) composed of LPS on one leaflet are shown for the first time. They are characterized with respect to their phase behavior, flip-flop of lipids and their usability to investigate the interaction with membrane active peptides or proteins. For the antimicrobial peptide LL-32 and for the bacterial porin OmpF the specificity of the interaction with asymmetric membranes is shown. The three reconstitution systems are compared with respect to their usability to investigate domain formation and interactions with peptides and proteins.

## Introduction

Since the introduction of the fluid mosaic model by SINGER and NICOLSON in 1972, the membrane itself and its unique, complex architecture has remained a central pillar of research today (Singer and Nicolson, 1972). Also in the early 1970s, KORNBERG and MCCONNELL were the first to successfully resolve the kinetics of lipid flip-flop between the individual membrane leaflets (Kornberg and McConnell, 1971). These two important hallmarks in research were essential steps in membrane biophysics. As a result, hitherto, various aspects, such as the molecular dynamics within the membrane, lipid specificity and the composition of the membrane in general, have entered the focus of current research and represent a discipline that combines classical physics, chemistry, biology, and medicine.

Many new insights into lipid membranes and their interaction with peptides and proteins have been gained by reconstituted membranes in biophysical experiments. These systems often accept the limitation that the model membranes usually have a symmetrical lipid architecture. There is a lively discussion about the existence and nature of lateral heterogeneity or domains, also known as lipid rafts, and their importance for the function of membrane processes. However, relatively little is known about the importance of axial heterogeneity or lipid asymmetry in membranes.

Within the manifold functionalities of cell membranes, asymmetry can explain various directed effects. Membrane asymmetry is induced and preserved in the form of variations of the headgroups and the length and degree of saturation of the hydrocarbon tail of the individual membrane components. A group of proteins, the flippases (outer leaflet to inner leaflet translocation of lipids) and floppases (inner leaflet to outer leaflet translocation of lipids) actively maintain the asymmetric lipid distribution (Marquardt et al., 2015; Rivel et al., 2019). Besides the axial heterogeneity referred to as membrane asymmetry, lateral membrane heterogeneity includes aspects such as lipid rafts, the formation of microdomains and lipid sorting. However, protein asymmetry is probably the most frequently investigated form of asymmetry on the cellular level. Surface proteins (that are attached to the membrane) or peripheral proteins (that partially penetrate the lipid bilayer) are often found accumulated on a certain membrane leaflet (Van Meer et al., 2008). Proteins involved in intracellular processes or in cellular defense mechanisms are primarily located on the inner leaflet or outer leaflet, respectively.

In recent years, the very limited range of reconstitution techniques suitable for the production of asymmetric membranes [including Montal-Mueller membranes (Montal and Mueller, 1972) and Langmuir-Blodgett films (Blodgett, 1934; Rinia et al., 1999)] has been supplemented by few but very useful additional techniques. These include the preparation of asymmetric GUVs by the phase transfer method (Pautot et al., 2003) and of smaller liposomes by cyclodextrin-mediated lipid exchange (Cheng and London, 2009). Thus, it is possible to characterize coupling or decoupling of lateral heterogeneity across the lipid bilayer, the effects of lipid asymmetry on protein localization, orientation, and conformation, and the influence of proteins on lipid distribution within a single leaflet and between the two leaflets (Van Meer et al., 2008). Simulations of distinct membrane models, e.g., for cancer and normal membranes, hint to a loss of asymmetry from healthy to unhealthy condition (Rivel et al., 2019). This loss of membrane asymmetry during disease shows the importance of this topic in the development of new drugs and therapeutic approaches.

In this study we focus on the asymmetry of the outer membrane of Gram-negative bacteria as the most asymmetric membrane described so far. We have modeled the asymmetric membrane with LPS being the main component in the bacterial outer leaflet and a phospholipid mixture (PL) as inner leaflet. At this point, it is noteworthy, that unlike eukaryotic membranes, the bacterial membrane has a much higher degree of flexibility, which is due to the packing density of the LPS in the outer leaflet. We modeled the phospholipid-rich inner leaflet from the three main components PE, PG and cardiolipin (CL), in a ratio equivalent to the composition of the cytoplasmic inner membranes (Gutsmann et al., 2015) of bacteria (Cronan and Vagelos, 1972; Osborn et al., 1972; Zachowski, 1993). Gram-negative bacteria feature two major forms of LPS – smooth (S) and rough (R) LPS (Alexander and Rietschel, 2001). Strains with S-form LPS harbor the full core oligosaccharide and O-antigen regions in the outer leaflet contrasting rough mutants expressing the glycolipid without O-antigen and truncated core oligosaccharide. For this project, we designed the outer leaflet with LPS R45 from the deep rough mutant of *Proteus mirabilis* strain R45. This LPS has a nearly conical molecular geometry shaped by full length lipid A and an inner core unit, that is composed of 3-deoxy-D-manno-2-octulsonic acid (Kdo) with a non-stoichiometric (approx. 50%) substitution of L-arabinose at two positions resulting in a reduced negative net charge (−3.0) (Wiese et al., 1998; Howe et al., 2007).

Within the scope of this paper, we elucidate the functions of the two membrane leaflets of LPS/PL membranes to analyze lipid asymmetry in bacterial membranes and to address the influence of this asymmetry on membrane permeability to drugs and small molecules. Therefore, we have chosen a set of biophysical techniques to investigate and compare the properties of free-standing (Montal and Mueller, 1972) and solid supported mono- and bilayers [Langmuir-Blodgett films (Blodgett, 1934)] and GUVs (Pautot et al., 2003). We provide clear evidence, that phase separation in bilayers can be different from the sum of the properties of the individual monolayers. With the help of fluorescence spectroscopy, i.e., dithionite quenching, lipid flip-flop rates were determined. Fluorescence microscopy of both symmetric and asymmetric vesicles showed the formation of LPS/LPS as well as LPS/PL domains within a narrow temperature range. The potency to reconstitute asymmetric membranes and to characterize membrane properties such as the flip-flop of lipids and the phase behavior across the lipid leaflets are essential parts of this work. The connection between phase separation and lipid flip-flop rates are yet not clear understood, but wide time ranges for lipid translocation were already described (Marquardt et al., 2015). The flip-flop rates discussed can range from a few seconds to hours or days depending on the lipid composition, membrane reconstitution system, fluorescent probe or temperature (Rosoff, 1996). In addition to the characterization of asymmetric membranes, we have used them to study their interaction with peptides and proteins. We show that the antimicrobial peptide LL-32 and the bacterial protein OmpF specifically interact with the two sides of asymmetric LPS/PL membranes.

## Materials and Methods

### Chemicals and Buffers

As buffer system, we used 100 mM KCl (potassium chloride), 5 mM MgCl_2_ (magnesium chloride), and 5 mM HEPES [4-(2-hydroxyethyl)-1-piperazineethanesulfonic acid] adjusted to neutral pH (pH = 7.0) with 1 M KOH (potassium hydroxide) TitriPUR (Merck, Darmstadt, Germany). The buffer was sterile filtered using a 0.22 μm pore size filter (ExpressTM PLUS, Millipore, Billerica, MA, United States) and stored at 4°C until use. For fluorescence quenching either KI (potassium iodide) or Na_2_S_2_O_4_ (sodium dithionite) was used. All chemicals were purchased from Sigma-Aldrich (Merck, Darmstadt, Germany). For the generation of asymmetric vesicles, pure anhydrous dodecan and a mixture of anhydrous dodecan and silicone oil (AR 200) in a ratio of 99:1 (v/v) was used. All solvents, including chloroform (CHCl_3_), ethanol (EtOH) and methanol (MeOH) were either purchased from Sigma-Aldrich (Merck, Darmstadt, Germany) or Acros Organics (Thermo Fisher Scientific, Rochester, NY, United States).

### Phospholipids and Lipopolysaccharides (LPS)

The inner membrane of Gram-negative bacteria was reconstituted from a PL-mixture composed of L-α-phosphatidylethanolamine from *E. coli* (PE, Avanti 840027P), L-α-phosphatidylglycerol from *E. coli* (PG, Avanti 841188P), and 1’,3’-bis[1,2-dioleoyl-*sn*-glycero-3-phospho]-glycerol (DPG, Avanti 710335P) also referred to as 18:1 cardiolipin (CL) in a lipid ratio of 81:17:2. This lipid mixture ratio is originally derived from the phospholipid composition of the inner leaflet of the OM of *Salmonella typhimurium* (Osborn et al., 1972; Shaw, 1974). The lipopolysaccharides used were extracted by the phenol/chloroform/petroleum ether method (Galanos et al., 1969) from a deep rough mutant of *Proteus mirabilis* strain R45. LPS R45 was fluorescently labeled with FITC by binding to the aminoarabinose (Ara4N). All phospholipids were purchased from Avanti (Avanti Polar Lipids Inc., Alabaster, AL, United States) and prepared at stock concentrations of 10 mg/mL in CHCl_3_ as described in earlier publications. Fluorescence conjugated lipids *N*-(7-nitrobenz-2-oxa-1,3-diazol-4-yl)-1,2-dihexadecanoyl-*sn*-glycero-3-phosphoethanolamine (NBD-PE) and Lissamine^TM^ Rhodamine B 1,2-dihexadecanoyl-*sn*-glycero-3-phosphoethanolamine (Rho-DHPE), were purchased from Life Technologies (Carlsbad, CA, United States). The lipid-dye conjugate FITC-phosphatidylethanolamine (FITC-PE) was purchased from Molecular Probes (Invitrogen GmbH, Darmstadt, Germany).

### Peptides and Proteins

The human cathelicidin fragment LL-32 with and without fluorescent Lissamine^TM^ Rhodamine B label were synthesized in-house (Research Center Borstel, Leibniz Lung Center, Germany) in accordance with a standardized solid-phase Fmoc-synthesis protocol at 0.1 mmol scale (Kent, 1988). Peptide synthesis was performed in an automated experimental lapse on preloaded Wang resin on a peptide synthesizer 433A (Applied Biosystems, Carlsbad, CA, United States). The crude peptide was purified by gradient controlled reversed-phase HPLC at 214 nm with MeCN/H_2_O supplemented with 0.1% trifluoroacetic acid (TFA, Riedel-de Haën, Seelze, Germany). After chromatographic purification on a Proteo 4 μm 80 Å 4.6 × 250 mm or Gemini Polar C18 4 × 3 mm LC-column, all peptides were lyophilized and further characterized by mass spectrometry. For fluorescence spectroscopy or microscopy, peptide stock solutions of 1 mg/ml in ultrapure water were prepared. All other peptide stocks were dissolved in 0.01% (v/v) TFA at concentrations of 2 mg/ml or 1 mM, respectively. The antimicrobial peptides LL-32 and ^Rho^LL-32 were used in this study to analyze their interaction with LPS and asymmetric vesicles.

For the expression of OmpF, the expression plasmid pGOmpF harboring the ompF gene of *E. coli* K12 was transformed into *E. coli* strain BL21(DE3)omp8. OmpF was purified as described by Lamichhane et al. (2013) and stored in 0.5% octyl-POE (n-octyl-polyoxyethylene; Alexis Biochemicals, Lausen, Switzerland).

### Fourier-Transform Infrared Spectroscopy (FTIR)

FTIR spectroscopy is generally performed on lipid multilayers. Multilayers are obtained by spreading multilamellar vesicles (MLVs). For the reconstitution of phospholipid-LPS MLVs, lipid species and glycolipid species were individually dissolved in a solvent mix composed of CHCl_3_/MeOH in a volumetric ratio of 9:1 (v/v) with a final concentration of 20 mM. Afterward, the lipids PE, PG, and CL were then mixed in a molar lipid ratio of 81:17:2. Half of this lipid mixture was supplemented with LPS R45 in a 1:1 ratio (v/v). The organic solvent was evaporated under nitrogen and resuspended in buffer (100 mM KCl, 5 mM MgCl_2_, 5 mM HEPES, pH 7.0) giving again a final concentration of 20 mM. The resulting suspension was sonicated (Branson, Sonifier, Cell disruptor B15) for one minute. MLVs were generated by running all samples through a two-step temperature cycle (30 min at 67°C and 30 min at 4°C), which was repeated three times. To measure the phase transition temperature (T_m_) 20 μl of the suspensions were placed between two calcium fluoride (CaF_2_) crystals (Korth Kristalle GmbH, Altenholz, Germany) with a 12.5 μm Teflon spacer. The samples were tempered in a temperature range of 10°C to 70°C with a heating rate of 2°C/min and the IR spectra were continuously recorded (IFS-55 spectrometer, Bruker, Karlsruhe, Germany). The phase transition was determined by the shift of the absorbance band of the symmetric stretching vibration of the methylene groups (ν = 2865-2845 cm^–1^) (Colthup et al., 1990). Experiments were repeated at least two times and representative curves are shown.

### Film Balance and Atomic Force Microscopy (AFM)

Lipid monolayers at the air/water interface were prepared by using a film balance. Measurements were performed on a KSV Nima Langmuir-Blodgett trough (Biolin Scientific Inc., Stockholm, Sweden). The temperature of the buffer/subphase was controlled by water circulation (Haake, Karlsruhe, Germany) and set to 21°C. The trough was filled with 65 ml buffer solution. Experiments were performed as follows: lipid solution PE/PG/CL (81:17:2) or LPS R45 solved in CHCl_3_ or CHCl_3_/MeOH 9:1 (v/v) were spread onto the surface of the subphase. After an equilibration time of 10 min at zero pressure, a pressure-area-isotherm was recorded by a symmetric compression of the effective film area with a constant compression rate of 10 mm⋅min^–1^. The monolayers were compressed to a lateral pressure of 20 mN/m which is in the range of the values discussed for the lateral pressure in cell membranes (Marčelja, 1974). The monolayers were equilibrated for 1 h at constant lateral pressure. For AFM measurements, monolayers were transferred onto mica plates according to the Langmuir-Blodgett technique with a pulling speed of 10 μm/s. For bilayer preparation, two individual monolayers were prepared and transferred one by one onto each other. Monolayers were imaged in air with the MFP-3D (Asylum Research, Santa Barbara, CA, United States) in AC-mode using NSG11 B cantilevers with a typical spring constant of 5.5 N/m and a resonance frequency of 150 kHz (NT-MDT, Moscow, Russia). Bilayers were imaged in buffer using CSG 11B cantilevers with a typical spring constant of *k* ∼ 30 mN/m and a resonance frequency of ω_0_ ∼ 9.8 kHz (NT-MDT, Moscow, Russia). For additional fluorescence imaging the LPS monolayer was labeled with 1% NBD-PE. The image processing was performed using the MFP-3D associated software package under IGOR Pro (Lake Oswego, OR, United States).

### Free-Standing Membranes

Planar bilayers were prepared according to the Montal-Mueller technique (Montal and Mueller, 1972) as described earlier (Wiese and Seydel, 2000; Gutsmann et al., 2015). Briefly, asymmetric bilayers were formed by opposing two lipid monolayers prepared on aqueous subphases from CHCl_3_ or CHCl_3_/MeOH solutions of the lipids or LPS, respectively, at a small aperture (∅∼150 μm) in a thin Teflon septum. The inner membrane of Gram-negative bacteria was reconstituted from a PL-mixture composed of PE, PG, and CL in a lipid ratio of 81:17:2. For electrical measurements, planar membranes were voltage-clamped via a pair of Ag/AgCl-electrodes (type IVM E255, Advanced Laboratory Research Inc., Franklin, United States) connected to the head-stage of an L/M-PCA patch-clamp amplifier (List-Medical, Darmstadt, Germany). All measurements were performed in 100 mM KCl, 5 mM MgCl_2_, 5 mM HEPES at 37°C and a pH-value of 7.0. For the fluorescence micrographs a home build microscope was used which is based on a mercury arc lamp (HBO 103 W/2, Osram, Munich, Germany), a dichroitic mirror (515DRLP, Omega Opticals, Brattleboro, VT, United States), an objective (M Plan APO 50x, Mitutoyo America Corporation, Aurora, IL, United States), two filters (455DF70 and 515ALP, Omega Opticals, Brattleboro, VT, United States), and a CCD-camera (C8484-02, Hamamatsu Photonics Germany GmbH, Herrsching, Germany). Both membrane sides, the PL as well as the LPS layer could be fluorescently labeled by adding 1% the lipid-dye conjugate NBD-PE. The proper bilayer formation was controlled by measuring the membrane current and capacitance. For apertures of about 150 μm the current was below 2 pA and the capacitance above 70 pF.

### Preparation of Asymmetric Vesicles

Outer and inner membrane leaflets were prepared in individual approaches and combined in the final centrifugation step. As inner leaflet a lipid mixture consisting of PE/PG/CL (81:17:2) is prepared first. For this purpose, all lipids were dissolved in CHCl_3_ to a final concentration of 2.5 mg/ml. From this lipid solution, 100 μL were transferred into a 5 mL screw-cap vessel and the solvent was evaporated under a stream of N_2_ (2 min/100 μl). After complete evaporation, 2 mL anhydrous dodecane were added to the lipids (final concentration 0.125 mg/mL). The solution was treated with sonication (Branson, Sonifier, Cell disruptor B15) for 30 min and then incubated over night at room temperature (T = 23°C). The next day, 250 μL buffer were added to the same solution and left to stir for 3 h at 300 rpm on a magnetic stirring plate (magnetic stirring fish of 10 × 6 mm). The phospholipids self-assemble as inverted micelles facing the headgroups into the aqueous phase of the small buffer droplets and the fatty acid chains into the hydrophobic dodecane phase. While the inner leaflet preparation is stirred, the outer leaflet preparation is performed: For this, LPS was solved in a mixture of anhydrous dodecane and silicone oil in a volumetric ratio of 99:1 (v/v) giving a final LPS concentration of 0.05 mg/mL. In a 50 mL Falcon^®^ tube (Corning^TM^ CentriStar centrifugation tubes), 1 mL of buffer solution was covered over by 2 mL of the LPS solution. The buffer contained 100 mM KCl, 5 mM MgCl_2_, 5 mM HEPES (pH 7.0). Because of the phase separation between the buffer and the dodecane:silicone oil the LPS arranged at the phase boundary with the headgroups orientated into the buffer solution and the fatty acid chains into the dodecane. The incubation time was about three hours. Afterward, 150 μL of the phospholipid emulsion from the inner leaflet preparation was gently pipetted on top of the LPS solution and centrifuged (120^∗^g; 10 min; 4°C). The final vesicle solution was removed by a 21 gauge syringe (B. Braun, Sterican^®^, 21 G x 1 1/2 ‘’) (Pautot et al., 2003). To proof the asymmetry, fluorescent dies were introduced in both leaflets. The LPS leaflet was labeled with 0.5% Rho-DHPE and the PL leaflet with 1% NBD-PE. By adding a fluorescence quencher, e.g., KI, the orientation of the fluorescent label as well as the integrity of the vesicles could be validated.

### Fluorescence Microscopy

Fluorescence microscopy was performed at 23°C either on a motorized inverted fluorescence microscope (IX-81, Olympus, Tokyo, Japan) or on a Leica TCS SP3 spectral confocal microscope (Leica Microsystems Heidelberg GmbH, Heidelberg, Germany). For confocal imaging, FITC labels were excited at λ_Ex_ = 488 nm, while the fluorescence emission was recorded from λ_Em_ = 500-550 nm. Vesicles were investigated without further dilution as (i) untreated samples, (ii) samples with added quencher (KI, 0.83 M) and (iii) samples with added quencher plus added peptide (^Rho^LL-32, 38 nM). In the presence of fluorescently labeled ^Rho^LL-32 excitation of FITC and Rho channels occurred back-to-back at 488 and 543 nm respectively, while the fluorescence was recorded from 500 to 550 nm and from 580 to 800 nm, respectively. In the recorded micrographs (Figure 4) FITC fluorescence was assigned to green color while Rho was assigned to red. To prove the existence of vesicles with quenched fluorescence, pictures with transmitted white light were recorded. For conventional fluorescence microscopy of aGUVs, images were acquired with an UplanSApo100xO oil-immersion objective (Olympus, Hamburg, Germany) in intX mode (intelligent exposure) at 23°C and collected with the software Cell^∗^P (Olympus Soft Imaging Solutions, Münster, Germany). The microscopy images (Figures 6C,D) were processed with Fiji (49, 50) (NIH, Bethesda, MD, United States) and subsequent evaluation by fluorescence dye distribution analysis (Figures 6c,d).

### Fluorescence Spectroscopy

To determine the permeabilization of asymmetric GUVs by the pore forming peptide LL-32 and the protein OmpF aGUVs were prepared of LPS R45 outside and the PL mixture inside, or *vice versa*. In both cases the inner leaflet was labeled with 1% NBD-PE. The fluorescence was measured by a spectrometer (Fluorolog 3, Horiba Jobin Yvon GmbH, Bensheim, Germany). aGUVs (1.5 mL) were used in the concentration obtained after preparation. The first addition after 10 min were 15 μL Na_2_S_2_O_4_ (1 M, end conc. 10 mM) or buffer as control. Second addition after 20 min were 15 μL LL-32 (3 mM, end conc. 30 μM), 15 μL OmpF (end conc. 5 μg/mL), or the respective solvent of LL-32 (0.01% TFA) or OmpF (0.004% octyl-POE). Experiments were repeated in three independent experiments (n = 3). Respective curves of the quotient between intensity after peptide/protein addition and intensity after buffer addition are shown.

## Results

### Characterization of the Phase Behavior of the Individual Components

**FIGURE 1:**
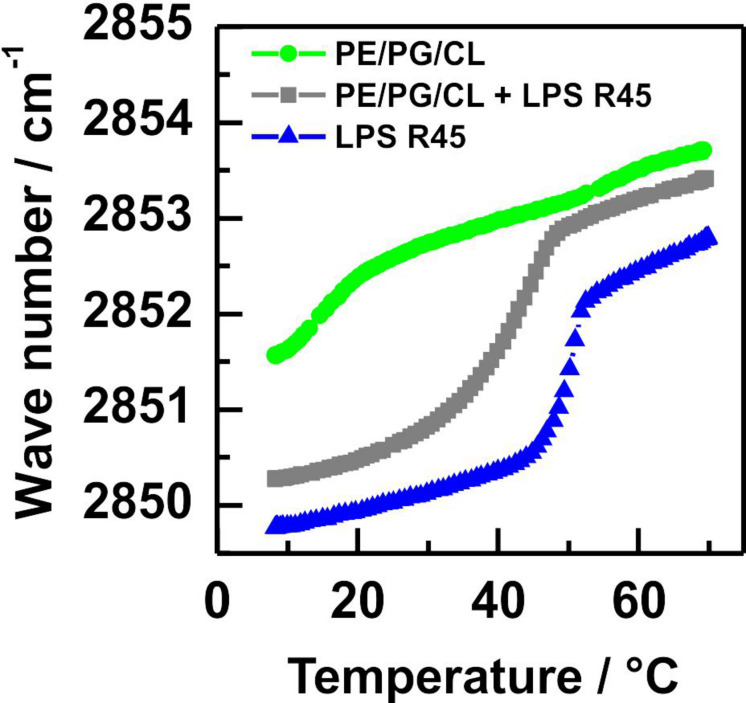
The equimolar mixture of LPS R45 and PL has a phase transition between those of LPS and PL alone. Phase transitions of membranes reconstituted from pure phospholipids (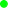), pure LPS R45 (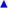), and an equimolar mixture of both (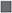) were recorded in FTIR measurements by measuring the symmetric CH_2_-stretching vibrations within a temperature window from 10°C to 70°C. The lipid mixture PE/PG/CL (81:17:2 w/w/w) shows a broad phase transition around 17°C, whereas for the pure LPS R45 a well-defined phase transition at 50°C is detected. The equimolar mixture of both, phospholipids and LPS exhibits a shifted phase transition to lower temperatures (T_m_ = 42°C) with a slightly less sharp characteristic. All experiments were performed with LPS and lipid concentration of 20 mM in 100 mM KCl, 5 mM MgCl_2_, 5 mM HEPES at pH 7.0.

Fourier transform infrared spectroscopy of symmetric MLVs. First, we characterized the phase behavior of the used lipids and lipid mixtures with the help of FTIR spectroscopy. We studied the induction of conformational changes in the stretching vibrations of the CH_2_-groups of the acyl chains over a temperature range from 10°C to 70°C for pure LPS and pure phospholipid membranes as well as a mixture of the two (Figure 1). For the pure PL mixture two main transitions at T_m__1_ = 15°C and T_m__2_ = 50°C were detected. These two phase transitions can correspond to the individual phase transitions of the different components in this lipid mixture (Figure 1, green line). However, already at room temperature of 21°C the wave number is around ν = 2852.5 cm^–1^, which is indicative for an almost fluid phase behavior. The pure LPS R45 of *P. mirabilis* showed a phase transition at T_m_ = 50°C (Figure 1, blue line). With an equimolar mixing ratio of PL and LPS only one phase transition (T_m_, _mix_ = 42°C) was detected, which had a smoother transition in-between the phase transitions of the pure LPS 45 and the PL mixture (Figure 1, gray line).

### Characterization of Asymmetric LPS/PL Lipid Membranes

**FIGURE 2:**
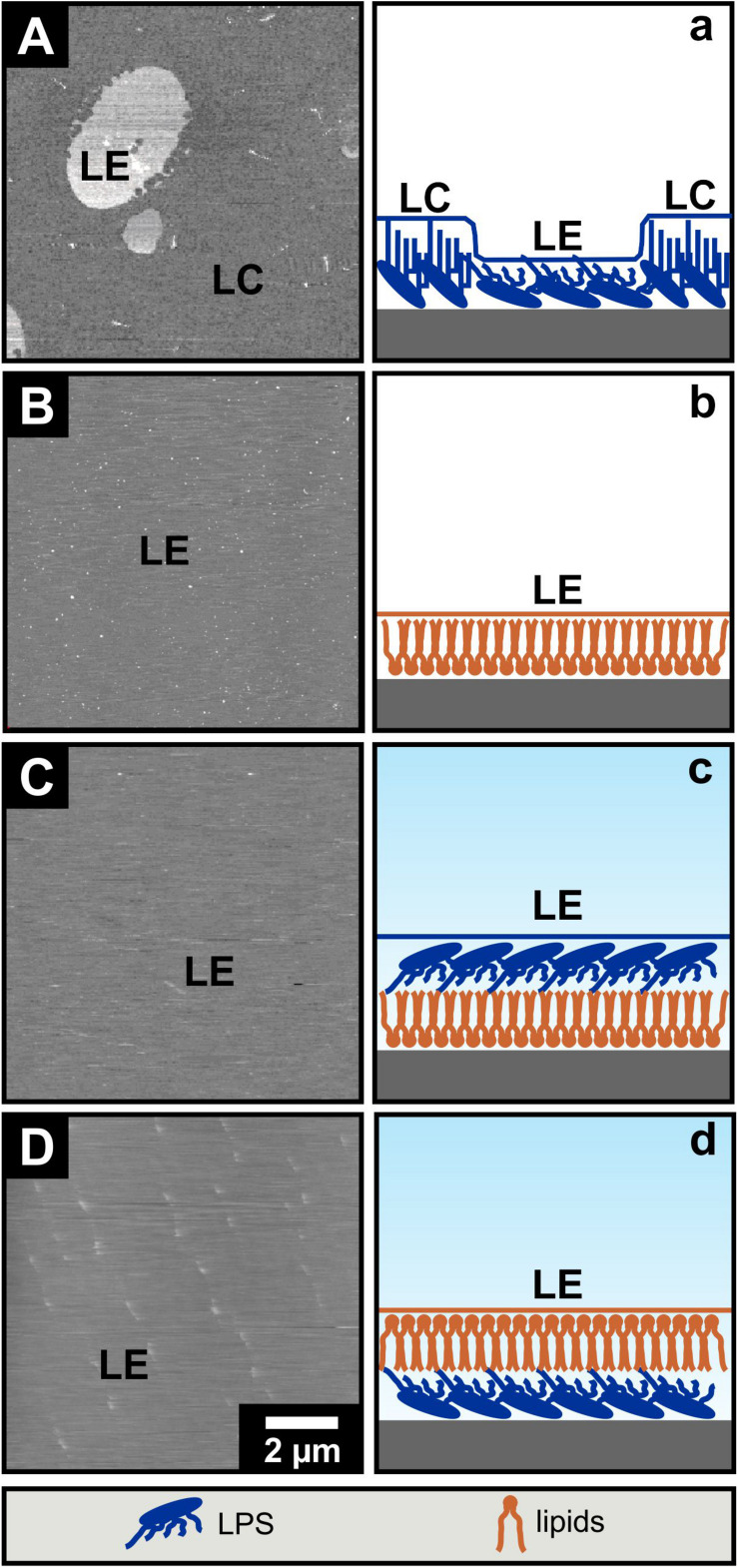
The phase separation of LPS R45 solid supported Langmuir-Blodgett monolayers is not maintained in asymmetric bilayers. Solid supported Langmuir-Blodgett monolayers **(A,B)** were imaged by AFM in air while imaging of Langmuir-Blodgett bilayers **(C,D)** was conducted in buffer. **(A,a)** LPS R45 monolayer; liquid expanded (LE) domains appear higher compared to the liquid condensed (LC) domains in the attractive regime of the AFM AC-mode; **(B,b)** PL (PE:PG:CL; 81:17:2; w/w/w) monolayer; **(C,c)** LPS R45/PL bilayer with the PL adsorbed on the solid support and the LPS R45 oriented to the buffer; and **(D,d)** PL/LPS R45 bilayer with the LPS adsorbed on the solid support and the PL oriented to the buffer. Only the LPS R45 monolayer showed a phase separation. Monolayer formation, Langmuir-Blodgett transfer and AFM imaging were performed in 100 mM KCl, 5 mM MgCl_2_, 5 mM HEPES buffer at pH 7.0 and T = 21°C. Lateral pressure of the monolayer during transfer to the mica was p = 20 mN/m. AFM height images are shown with a gray scale range of 5 nm.

AFM imaging of solid supported mono- and bilayers prepared by Langmuir-Blodgett transfer. In order to model biological membranes, one approach is the lipid deposition of Langmuir-Blodgett (LB) films on hydrophilic mica. Therewith solid supported lipid mono- and bilayers are generated in an either symmetric or asymmetric organization. Monolayers were prepared via the Langmuir-Blodgett technique onto the solid support and were subsequently imaged by AFM in air. The liquid expanded domains of an LPS R45 monolayer (Figure 2A) appear higher in the height images (Roes et al., 2005). Because of their more flexible fatty acid chains the liquid expanded (LE) domains are about 0.8 nm lower compared to the liquid condensed (LC) domains when imaged in contact mode (Figure 2a). The lateral size of the domains depends on the temperature and the lateral pressure. Under the same conditions no phase separation could be observed for the pure PL mixture (Figure 2B). To verify this result, a lipid-dye conjugate (1% NBD-PE) was added to the organic phase while preparation of the lipid solution. In doing so, the solid supported monolayer showed a homogenous fluorescence. This shows that the monolayer was in the fluid phase before Langmuir-Blodgett transfer (Roes et al., 2005). In another experiment, a second monolayer is prepared and transferred over the first. Thus, a bilayer is obtained, which can be altered in composition and orientation of the individual membrane components contributing to this bilayer. Bilayers were imaged in buffer. For asymmetric bilayers, two different preparation approaches were followed: The PL-mix is adsorbed on mica first and then coated with the LPS layer so that the LPS-containing layer is aligned toward the buffer (Figure 2C) or *vice versa* (Figure 2D). In both cases no phase separation was detected. In Figure 2D small objects can be seen which originate from free-flowing particles in the buffer. Again, the verification experiment with incorporation of NBD-PE into the bilayers resulted in a homogenous fluorescence distribution across the membrane in both scenarios (data not shown). All experiments were performed at room temperature (approx. 21°C).

**FIGURE 3:**
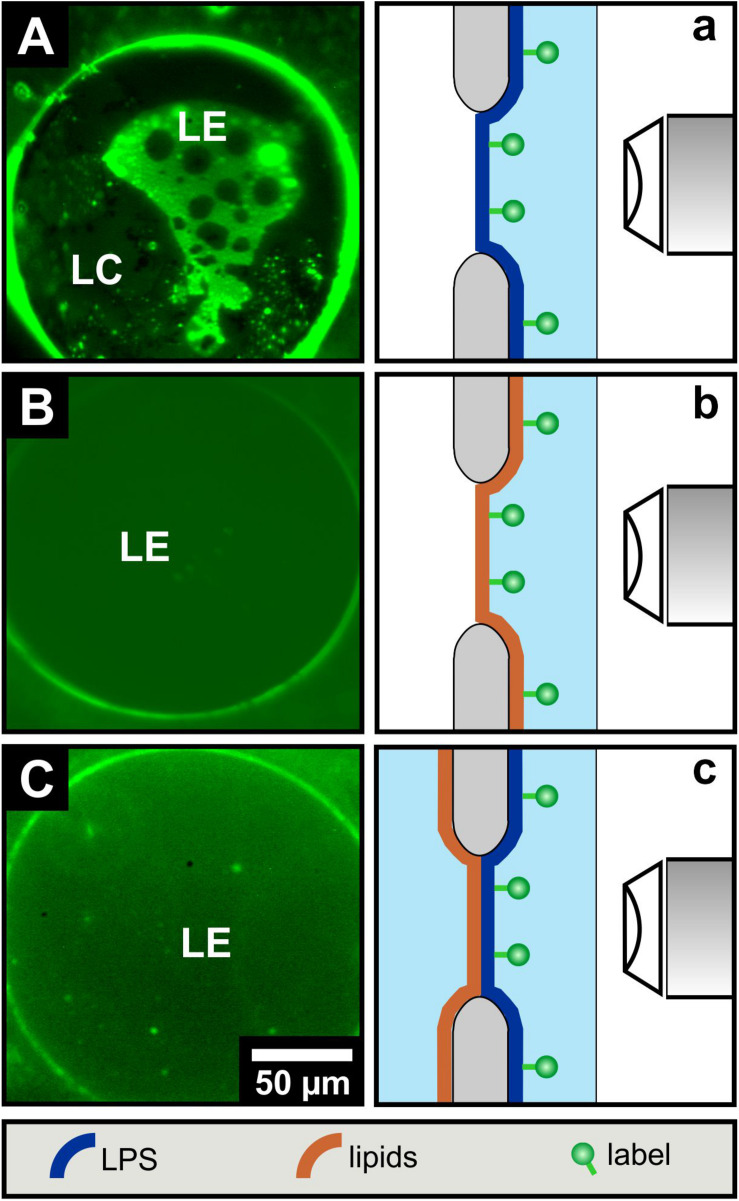
LPS R45 free-standing planar monolayers lose their phase separation upon combination to an LPS R45/PL free-standing bilayer. Fluorescence microscopic images of **(A)** a LPS R45 monolayer, **(B)** a PL (PE:PG:CL; 81:17:2; w/w/w) monolayer, and **(C)** a LPS R45/PL planar bilayer reconstituted over an aperture. For fluorescence labeling of an individual membrane leaflet, the lipid monolayer was supplement with 1% NBD-PE which partitions into the liquid expanded (LE) domains. As with the solid-supported membranes, phase separation in free-standing lipid systems occurred only for the LPS R45 monolayer. Experiments were conducted in 100 mM KCl, 5 mM MgCl_2_, 5 mM HEPES at pH 7.0 and T = 37°C.

Fluorescence microscopy of free-standing planar mono- and bilayers prepared by the Montal-Mueller technique. With the solid supported lipid systems, we could not clarify to what extent the opposite monolayers influence their phase behavior. As solid supports can change the phase behavior of lipid monolayers and bilayers, we have investigated asymmetric free-standing planar lipid bilayers to complement the data obtained so far. LPS R45 as well as the PL mixture were each mixed with 1% NBD-PE with a preference to the fluid phase. This unequally distributed fluorescent-lipid conjugates into the fluid membrane domains leading to an enhanced green fluorescence. The fluorescence dye is excluded from the more rigid membrane areas as those domains appear black. In a first series of experiments only LPS R45 monolayers were reconstituted over the aperture of the Teflon foil and fluorescence images were taken (Figure 3A). The physiological body temperature of healthy humans of about 37°C was used for the free-standing membrane experiments to obtain stable LPS R45 monolayers during the process of preparation. At this temperature, a phase separation of the LE and LC domains could be observed. Progressing heating of the monolayer to 42°C (resembling fever) resulted in a fluidization of the monolayer and therewith a phase melting. The original phase separation was lost at 42°C (SI, Supplementary Figure S1). This effect was reversible. Compared to the LPS monolayer, both the pure PL monolayer and the full reconstituted asymmetric LPS R45/PL planar bilayer remain in the LE phase showing no domains (Figures 3B,C). In Figure 3C small objects can be seen which originate from free-flowing particles in the buffer. Even by cooling, it was not possible to reach a temperature at which a phase separation in the bilayer was induced. Temperatures below 25°C led to a loss of membrane stability and thus the asymmetric LPS-containing bilayers ruptured. Further, it was not possible to generate two individual LPS R45 monolayers on one another for the reconstitution of a symmetric free-standing LPS-LPS bilayer. This instability might be due to steric hindrance and spatial arrangement of the hydrocarbon chains of the LPS. Another reason could be the unnatural high rigidity of such a membrane that cannot be compensated by the more fluid PL leaflet. The asymmetric distribution of LPS and PL on both leaflets leads to differences in the Gouy-Chapman, the dipole and the Born potential. These three potentials on both sides of the bilayer sum up to the overall inner membrane potential. In case of an asymmetric membrane an inner membrane potential difference exists which can be determined by using the inner field compensation (IFC) method. This method provided evidence on a stable asymmetric lipid distribution over the membrane’s lifetime of up to two hours (Hagge et al., 2004).

**FIGURE 4:**
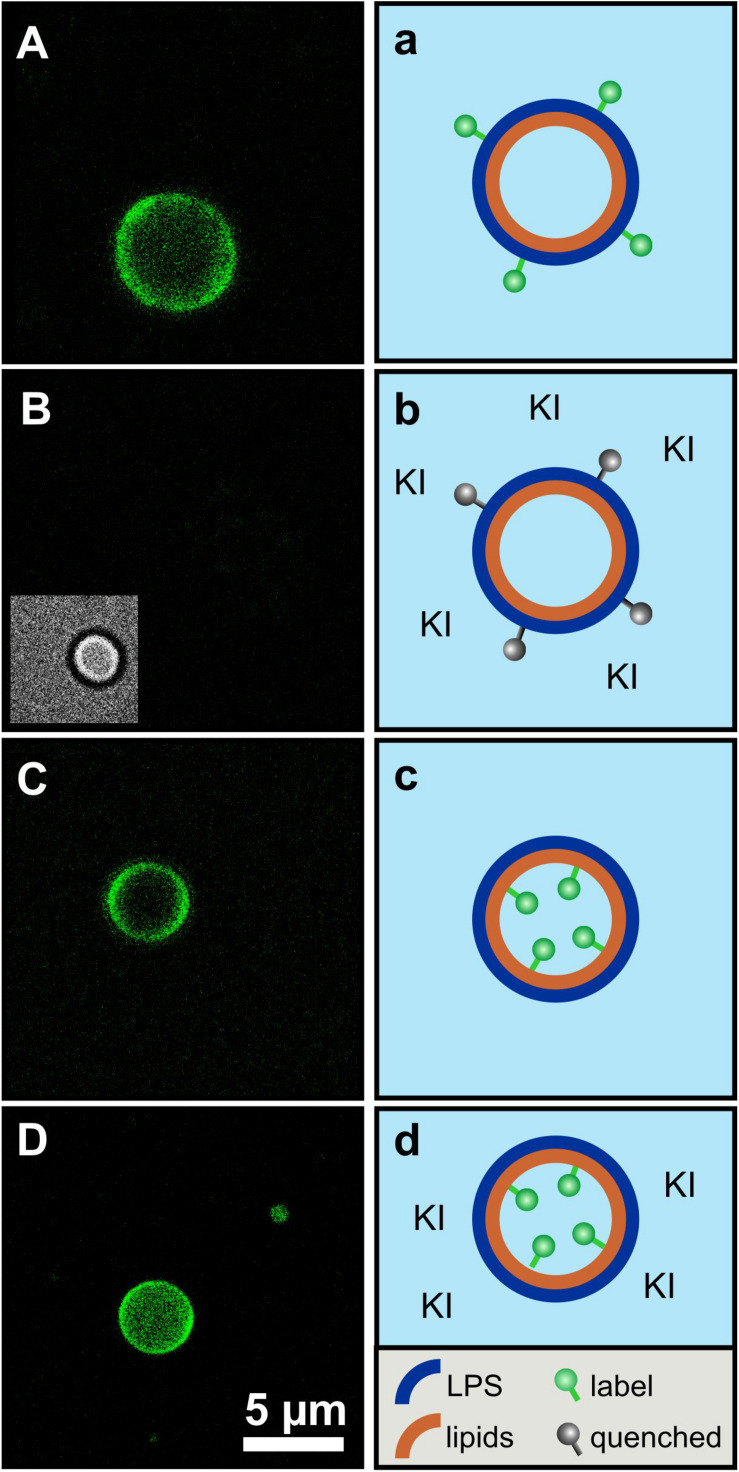
The asymmetry of giant unilamellar LPS R45/PL vesicles is confirmed by leaflet-specific fluorescence quenching with KI. **(A,a)** Asymmetric vesicles with LPS R45 in the outer leaflet and PL (PE:PG:CL; 81:17:2; w/w/w) in the inner leaflet; 5% (w/w) of the LPS R45 molecules in the outer leaflet were fluorescent FITC-LPS R45; **(B,b)** Quenched fluorescence of the vesicles after the addition of KI. The inlet shows the vesicle in phase contrast; **(C,c)** Asymmetric vesicles with LPS R45 in the outer leaflet and PL in the inner leaflet; 2% (w/w) of the PL molecules in the inner leaflet were fluorescent FITC-PE; **(D,d)** Vesicles after the addition of KI. The fluorophores in the inner leaflet were protected from quenching. Experiments were carried out at 25°C in 100 mM KCl, 5 mM MgCl_2_, 5 mM HEPES buffer at pH 7.0 using a Leica TCS SP3 confocal microscope. Final KI concentration was 0.83 M; Scale bar applies to all images.

**FIGURE 5:**
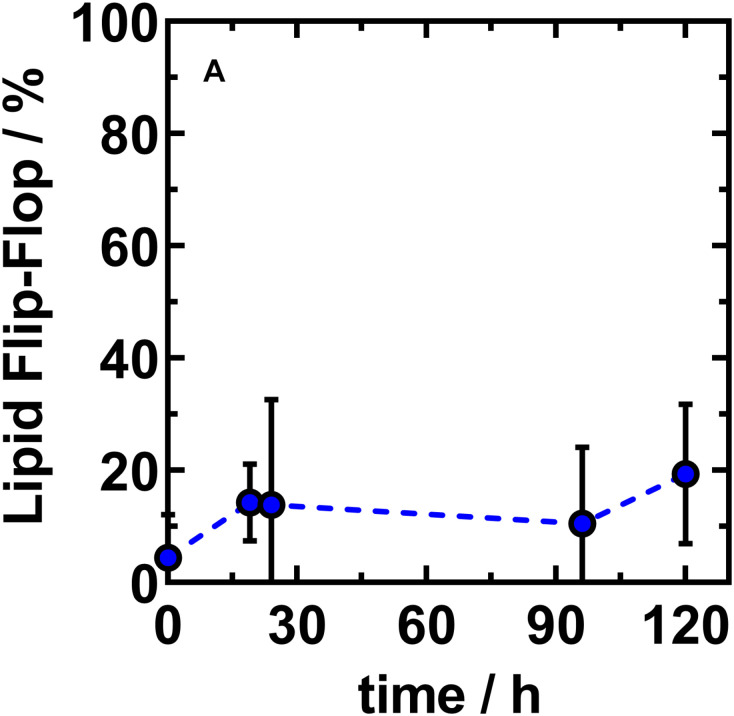
The net lipid translocation into the outer leaflet of asymmetric LPS R45/PL vesicles largely finds a saturation level at a NBD-PE fraction of about 20%. Fraction of NBD-PE found in the outer leaflet of asymmetric vesicles reconstituted from LPS R45 (OL) and PE:PG:CL (IL; 81:17:2; w/w/w). The ability of lipid flip-flop of NBD-PE is monitored over time. Initial flip-flop from inner to outer leaflet occurs fast (1 h) after phase transfer and asymmetric vesicle generation. Asymmetric vesicles with stable outer leaflet composition are achieved approximately 19 h after final preparation. Data shown represent the mean value of three independent experiments ± SD (*n* = 3).

**FIGURE 6:**
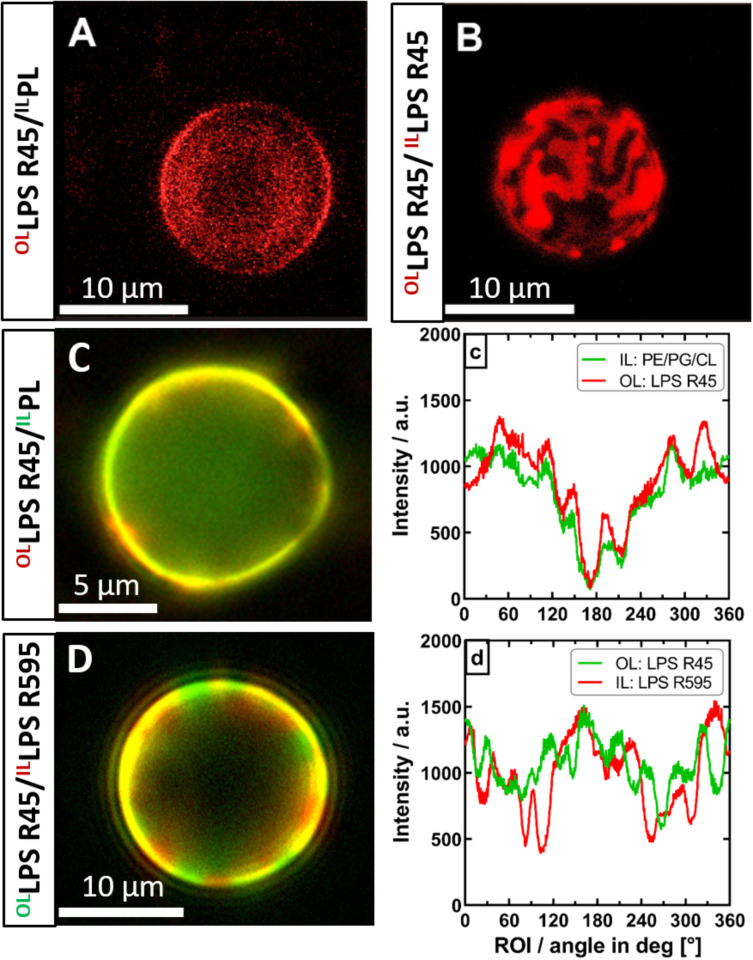
The phase separation of the outer leaflet of asymmetric vesicles is depending on interactions with the inner leaflet. Both asymmetric and symmetric giant unilamellar vesicles were generated by the phase transfer method. **(A)** Asymmetric LPS R45/^Rho^PL vesicles expose no phase separation (PL refers to PE:PG:CL; 81:17:2; w/w/w). **(B)** Symmetric ^Rho^LPS R45/^Rho^LPS R45 vesicles show dominant phase separation in the outer as well as in the inner leaflet (IL), exposing stripe and patch shaped domains. **(C)** Phase separation in asymmetric LPS R45/PL vesicles could not be enforced by lowering divalent cation content. **(c)** Fluorescence dye distribution analysis of IL and outer leaflet (OL). The fluorescent dye conjugates of both leaflets correlate and show an intensity minimum at approx. 180°. **(D)** Asymmetric ^FITC^LPS R45/^Rho^LPS R595 vesicles show a dominant phase separation with areas wherein the fatty acid chains of the different LPS types match (yellow) and mismatch (red/green). **(d)** Fluorescence dye distribution analysis of the individual LPS leaflets giving areas of correlation and anti-correlation of the different fluorescence labels. All experiments were run out in 100 mM KCl buffer supplemented with either 5 mM MgCl_2_ and 5 mM HEPES **(A+B,D)** or 0.5 mM CaCl_2_x2H_2_O **(C)** at pH 7. Confocal laser scanning microscopy **(A+B)** was performed at 26°C. Images were taken with a Leica SP3. Z-series were performed and compiled for 3D visualization. Inverse fluorescence microscopy was performed at room temperature with an Olympus IX-81.

Fluorescence quenching of asymmetric GUVs generated by phase transfer. For an even closer mimicry of the biological system, asymmetric vesicles were designed with a membrane curvature resembling bacterial cells. We used a protocol based on the original method as described by PAUTOT et al. (Pautot et al., 2003). We were successful to generate asymmetric vesicles with a full LPS outer leaflet and a full PL inner leaflet by the so-called phase transfer method. In addition, our protocol allowed us to generate for the first time inside out asymmetric vesicles with a full PL outer leaflet and a full LPS inner leaflet to gain accessibility from both sides. Furthermore, it is also possible to produce symmetric vesicles consisting of two pure LPS leaflets. The yield of the vesicles generated via phase transfer is significantly lower compared to other vesicle preparation methods. The yield is strongly dependent on the quality of lipid suspension during the preparational process. The vesicle size varies between two and 20 μm in diameter. Different approaches were followed for checking the overall asymmetric distribution: (i) Fluorescent labeled vesicles with LPS R45 containing 5% FITC-LPS R45 in the outer leaflet were prepared. After addition of an aqueous solution of potassium iodide (KI), the fluorescence of the vesicles was quenched due to the accessibility of the fluorophore in the outer leaflet (Figure 4A: before addition of KI, Figure 4B: after the addition of KI); (ii) In parallel, phase contrast images were taken to check whether the vesicle integrity was disturbed by the addition of or by the KI itself (inlet Figure 4B). This proved that the vesicles remained intact and the fluorescence quenching was successful, which demonstrates an exclusive localization of LPS in the outer leaflet. Since KI is classified as permeable to membranes due to its size and substance properties, a further experimental approach was needed. The aim was to test whether the observed effect is due to the outward orientation of the fluorophores or was possibly caused by membrane permeable quencher. Therefore, (iii) asymmetric vesicles with FITC-PE in the inner leaflet were prepared (Figure 4C). The addition of KI to the FITC-PE vesicles showed no effect and a continuous, stable fluorescence (Figure 4D) which indicates that the membrane integrity is strong and impermeable to the quencher compared to conventional phospholipid-only membranes. This also demonstrates that in a time frame of 5 days no significant amounts of phospholipids flipped from inner to outer leaflet (Figure 5). In a few cases, lipid domains could be detected at room temperature (Figure 6C), which was not the case for temperatures above 25°C.

After successful confirmation of the asymmetric membrane structure, we wanted to find out if lipid flip-flop in LPS-containing asymmetric vesicles is possible at all. In a further quenching approach, aGUVs that resembled the natural Gram-negative membrane model were prepared. The inner PL layer was additionally enriched with a lipid-dye conjugate of the same lipid species (1% NBD-PE). By means of fluorescence spectroscopy we exposed the prepared aGUVs to sodium dithionite as quencher for a period of up to 5 days. By repeated measurements at different points in time (Figure 5) we could confirm that a lipid exchange from the inner PL layer to the outer LPS layer takes place. On the other hand, after 120 h, saturation was reached in which a maximum of ∼20% of the fluorescence-labeled lipid species in the outer layer could be detected due to progressing quenching. The sometimes strongly varying percentages are due to the size distribution of the prepared vesicles, since there is a certain degree of heterogeneity due to the phase transfer method. The given maximum of 20% shows that if there is a flip-flop it cannot be higher that 20%; however, the decrease in fluorescence can also originate from a general leakage of some of the GUVs. Based on these data, we wanted to understand to what extent there is a connection between the lipid flip-flop and the microscopically visualized phase separation at temperatures below 25°C. More precisely, what happens first: Lipid flip-flop with subsequent phase separation or is there a phase separation already existing after aGUV preparation and the observed lipid flip-flop is created to compensate for other effects, such as membrane rigidity. To answer these questions, however, we looked at aGUVs in natural arrangement (LPS/PL), in inverse arrangement (PL/LPS), and uniform arrangement (LPS/LPS) microscopically. In order to be able to record effects between the individual layers, we used an approach in which both sides of the membrane were mixed with the same lipid-dye conjugate (Rho-DHPE) and an approach in which the inner and outer layers were provided with different dyes (1% NBD-PE and 0.5% Rho-DHPE). Figure 6 shows the results of those approaches. The structure of the ^Rho^LPS R45/PL membrane mimicking the natural composition does not show any separation (Figure 6A). We see an even fluorescence distribution over the z-stacks of these aGUVs. The situation becomes interesting when uniform ^Rho^LPS R45/^Rho^LPS R45 (Figure 6B) GUVs are viewed under the microscope: Here, there is a pronounced phase separation within the two layers in a registered manner (Figure 6B). At temperatures below 25°C phase separation in LPS/PL membranes could be observed in both leaflets of the ^Rho^LPS R45/^NBD^PL in a registered manner (Figures 6C,c). To check weather this effect is PL specific we prepared aGUVs composed of LPS extracted from different bacterial strains. Again, a strong phase separation was achieved when LPS layers of different species are prepared against each other (Figures 6D,d). We also detected a domain formation, but in an anti-registered manner in the individual layers of ^NBD^LPS R45/^Rho^LPS R595 aGUVs (Figure 6D).

### Interaction of Asymmetric GUVs With Pore-Forming Peptides and Proteins

The usability of asymmetric GUVs to investigate the activity of pore-forming peptides or proteins and the importance of the asymmetric composition is demonstrated in the following by microscopic and spectroscopic experiments.

**FIGURE 7:**
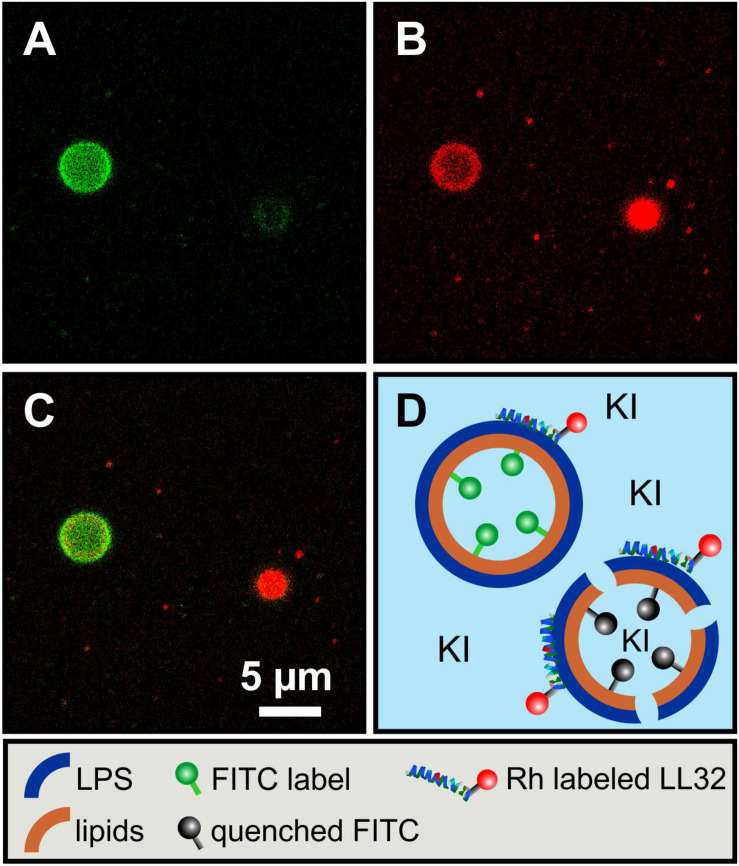
Fluorescence quenching in asymmetric LPS R45/PL vesicles allows the observation of a concentration dependent permeabilization by antimicrobial peptide LL-32. For fluorescent labeling of the vesicles 2% (w/w) of the PL (PE:PG:CL; 81:17:2; w/w/w) in the inner leaflet were fluorescent FITC-PE. Images show the vesicles after incubation with 0.83 M KI and 38 nM ^Rho^LL32. **(A)** FITC-PE fluorescence; **(B)** ^Rho^LL-32 fluorescence; **(C)** Superposition of **(A)** and **(B)**; **(D)** Illustration of the experiment. The left vesicle had taken up less ^Rho^LL-32, remained intact and therefore exhibited unquenched FITC fluorescence from its inner leaflet. The right vesicle had taken up more ^Rho^LL-32, was permeabilized and partly quenched by invading KI. Experiments were carried out at 25°C in 100 mM KCl, 5 mM MgCl_2_, 5 mM HEPES at pH 7.0 using a Leica TCS SP3 confocal microscope. Scale bar applies for **(A–C)**.

Fluorescence microscopy of asymmetric GUVs. First, we demonstrate the interaction between the asymmetric vesicles and the fluorescently labeled antimicrobial peptide ^Rho^LL-32 by using confocal microscopy. This peptide can induce lesions and at higher concentrations it can permeabilize the membrane. We used asymmetric vesicles labeled with FITC-PE in the inner leaflet and added KI to the solution. ^Rho^LL-32 was added, and the fluorescence of both fluorophores was imaged (Figure 7). After peptide addition some vesicles showed a weak fluorescence of ^Rho^LL-32 and still a strong fluorescence of the inner leaflet (Figure 7C, left vesicle). This demonstrates that only a limited number of peptides have attached to the outer lipid leaflet. The required threshold concentration of LL-32 was not reached, which prevented the formation of pores. Due to the lack of pore formation, the quenching molecule was unable to permeate the membrane and thus quench the fluorescence of the inner leaflet. If the concentration of ^Rho^LL-32 on the surface was higher (Figure 7C, right vesicle), the peptides could form pores leading to a permeabilization of the bilayer and a resulting decrease in the fluorescence of the FITC-PE. At higher peptide concentrations the GUVs were destroyed. This confirms that above a certain critical concentration, ^Rho^LL-32 induces lesions large enough to allow the quencher to access the inside of the vesicles. The absolute peptide concentration can have significant variations within the sample because of the small volumes added on the microscopy slide. Therefore, these data cannot be used to determine the absolute concentration necessary to induce membrane permeabilization. Compared to other experiments the LL-32 concentration of 38 nM was very low.

**FIGURE 8:**
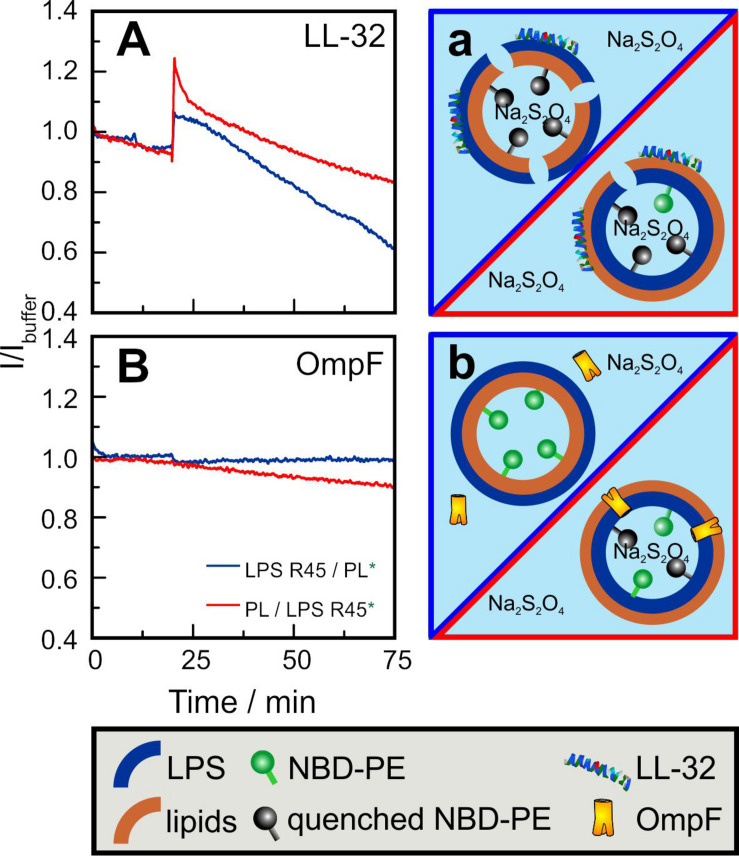
Inner leaflet labeled asymmetric GUVs enable the determination of orientation-sensitive quencher-uptake dynamics through **(A,a)** LL-32 induced lesions and through **(B,b)** bacterial porin OmpF. GUVs prepared by the phase transfer method were composed of LPS R45 on the outer leaflet and the PL mixture (PE:PG:CL; 81:17:2; w/w/w) on the inner leaflet (**A:** blue curves; **a**:blue framing) and *vice versa* (**A:** red curves; **a:** red framing). After 10 min, Na_2_S_2_O_4_ was added to a final concentration of 10 mM. **(A)** After 20 min, LL-32 was added to a final concentration of 30 μM. After a first intensity increase because of peptide insertion a decrease because of the quenching of the inner leaflet marker NBD-PE (2%) can be seen. **(B)** After 20 min OmpF was added to a final concentration of 5 μg/ml. Influx of the quencher Na_2_S_2_O_4_ only occurs if the PL mixture is faced outward and directly exposed to the protein. Experiments were performed in 100 mM KCl, 5 mM MgCl_2_, and 5 mM HEPES at pH 7.0 and at T = 37°C.

Fluorescence spectroscopy of asymmetric GUVs. In a second approach, we used fluorescence spectroscopy to show that LL-32 and the porin OmpF interact differently with the different leaflets of the asymmetric GUVs. After the addition of LL-32 to aGUVs with NBD-PE labeled inner leaflet, a quick increase in fluorescence was observed for both membrane compositions LPS R45/PL (natural outside-out orientation) and PL/LPS R45 (inside-out orientation) (Figure 8A). This can be caused by the insertion of LL-32 into the lipid bilayer leading to a change in the fluorophore lateral organization, changes of aggregation of LL-32, and of further unknown artefacts. The effect of LL-32 and other fragments of the human cathelicidin on liposomes including the permeabilization and the increase in their diameter as determined by DLS experiments was described in detail before (De Miguel et al., 2019). After this initial step, the fluorescence decreased because of the permeation of the quenching molecules (Na_2_S_2_O_4_) into the aGUVs. This effect was more pronounced for membranes in which the LPS R45 was present in the outer leaflet. Because of the higher number of negative charges in the LPS compared to the PL mixture the interaction with the polycationic LL-32 was stronger and led to a more intensive permeabilization. Opposite to this effect it has been shown that porins intercalate only from the PL side into an asymmetric LPS/PL membrane, but not from the LPS side (Hagge et al., 2002). Exactly this effect was also observed in the presented results (Figure 8B). After addition of OmpF to aGUVs composed of LPS R45 on the outer leaflet no change in the fluorescence intensity was detected. Addition to inside-out aGUVs (lipid mixture as outer leaflet) led to a linear decrease in intensity. Unlike LL-32, OmpF molecules did not destabilize the overall membrane integrity, but allowed a continuous permeation of Na_2_S_2_O_4_ molecules, which explains the slower decrease in fluorescence.

## Discussion

After Singer and Nicolson (1972) published their fluid mosaic model in 1971, M. BRETSCHER discussed the asymmetry in lipid membranes (Bretscher, 1972). Since then, a number of studies have shown how the lipid composition differs in the two leaflets of various membranes, what influence this has on the function (Gupta et al., 2020), localization and orientation of proteins, how asymmetry is established and maintained by active processes and which consequences changes in asymmetry can have. Despite all these advances, relatively little is still known about the underlying molecular processes. In recent years, techniques have been established to reconstitute asymmetric membranes that allow detailed characterization. In 2003, PAUTOT et al. presented a method by which the design of an asymmetric membrane was facilitated by phase transfer of two individual prepared lipid compartments (Pautot et al., 2003). Based on this approach, we developed a protocol that allowed the generation of asymmetric LPS-containing membranes in a size range between 2 μm to 20 μm. This size range resembles the size of living bacteria and enables the microscopic analysis of asymmetric membranes without solid support. For the first time, it was possible to prepare asymmetric vesicles with a pure LPS outer (natural) or even inner leaflet (unnatural) and phospholipids in the opposing leaflet. Furthermore, we can design vesicles composed of LPS on both leaflets with either LPS received from the same origin giving symmetric LPS vesicles with a uniform fatty acid chain distribution or LPS received from different origins giving asymmetric LPS vesicles with a non-uniform fatty acid chain distribution. In this study we used LPS purified from a deep rough mutant of *P. mirabilis* R45. This LPS preparation consists of a natural mixture of different number and length of fatty acids and other non-stoichiometric substituents. Therefore, domain formation driven by the different chemical species can occur. Furthermore, the phospholipids used to reconstitute the inner leaflet are a mixture of naturally occurring lipid species in PE, PG, and CL. The goal of this work was to generate the LPS/PL aGUVs, to compare their properties with those of planar lipid bilayers and solid supported membranes, to characterize their phase behavior, and to use them to analyze the lipid-dependent activity of the antimicrobial peptide LL-32 and the bacterial porin OmpF.

As early as, Brandenburg and Seydel (1990) reported for all enterobacterial LPS a gel to liquid crystalline phase transition occurring within a temperature range from 30°C to 37°C (Brandenburg and Seydel, 1990; Brandenburg et al., 1990) in buffer containing no divalent ions such as magnesium or calcium. The phase transition temperature for the herein used LPS R45 matches with around 35°C the literature values (Howe et al., 2007). Here, we used a buffer containing 5 mM MgCl_2_. This increases the stability of the membranes, i.e., that of the planar lipid bilayers and it increases the phase transition temperature to 45°C. The PL mixture has a phase transition temperature below 20°C and the mixture of LPS and PL of 40°C (Figure 1).

The phase separation in LPS monolayers (Figures 2A, 3A) can be explained easily. At 21°C and a lateral pressure of 20 mN/m most of the LPS molecules on the film balance were in a liquid condensed (LC) phase and some were in the liquid expanded (LE) phase. This led to a phase separation of the molecules (Figure 2A). This is in good agreement with the data obtained from the LPS monolayer prepared during the Montal-Mueller reconstitution of a planar membrane at 37°C (Figure 3A). The phospholipid molecules in the monolayers were all in the LE phase at 21°C (Figures 2B, 3B) and showed therefore no phase separation. These results are consistent with that obtained from the FTIR experiments using liposomes. Now, the interesting part of the measurements was the behavior of the bilayers. The results of the experiments showed that there was no phase separation of the LPS molecules in asymmetric LPS/PL bilayers on a solid support (Figures 2C,D) or prepared as a planar lipid bilayer (Figure 3C). The two layers influenced each other by shifting the phase transition temperature of the LPS to lower temperatures. In case of the solid supported bilayers it did not matter whether the LPS or the PL mixture was oriented toward the mica.

By using the phase transfer method to reconstitute asymmetric GUVs, we prepared asymmetric vesicles composed of LPS in the outer and phospholipids in the inner leaflet for the first time. Maktabi et al. (2019) published in 2019 a method to produce asymmetric LPS-containing vesicles as water-in-oil-in-water double emulsions prepared by a microfluidics approach. Attempts to reconstitute asymmetric LPS-containing vesicles by cyclodextrin-catalysis (Doktorova et al., 2018) did not work out so far. We could show that the prepared vesicles were asymmetric and stayed asymmetric for more than 5 days. The average diameters of the prepared vesicles were about 5 μm and therefore they could be used as a good bacterial membrane model in different experimental setups. The size of the aGUVs can be adjusted by modifying the parameters of the preparation of the buffer droplets and by changing the ion concentrations of the buffer. To proof the asymmetry, we determined the potency of potassium iodide to quench the fluorescence of dyes which were in the LPS or in the phospholipid leaflet (Figure 4). Using fluorescence quenching, we have shown that the vesicles generated by phase transfer are indeed asymmetric and at the same time impermeable to potassium iodide. In addition, we have successfully demonstrated that the asymmetry remained stable over days. Therefore, we studied the lipid flip-flop rates. Bearing in mind that phospholipid flip-flop strongly depends on headgroups with a smaller dependence on acyl chain length and that under physiological pH (pH 7.4) flip-flop rates increase in the order of PC < PG < PA < PE, we carried out our experiments on a cuvette scale, measuring the lipid flip-flop of fluorescence-labeled PE. In general, rates measured for PE are at least 10-times greater than those of PC (Rosoff, 1996). In the literature, large activation energies for flip-flop ranging from 38 kcal/mol for the longest acyl chain derivative of PC to 25 kcal/mol for the PE derivatives could be found (Rosoff, 1996). An increase in acyl chain length is linked to reduced phospholipid flip-flop rates whereas acidification, e.g., change in pH from pH 7.4 to 4.0 which is described for phagosome acidification, accelerate lipid flip-flop by 500-fold. The most frequent obstacle in the determination of lipid flip-flop is probably the strong dependence of the insolubility of the polar head groups in the intracellular space or the membrane interior, respectively. The flip-flop rate in our NBD-PE labeled GUVs was below 20% within five days (Figure 5). This was the flip-flop of the fluorescently labeled PE which might be reduced compared to unlabeled phospholipids. However, we propose that the flip-flop of the even larger LPS molecules composed of four glucans would not be higher. This is supported by the observation that the FITC-labeled LPS was exclusively in the outer leaflet after five days (data not shown). On a shorter time scale (up to 2 h) we investigated the asymmetry of LPS/PL planar membranes by measuring the inner membrane potential difference by the inner field compensation method (Hagge et al., 2004). Even within the first seconds and minutes the asymmetry of the bilayers was constant.

A decisive property for lipid membranes is the phase behavior and thus the lateral diffusion of lipids and proteins in the membrane. Differences in the lateral diffusion between the two leaflets have been shown by fluorescence imaging microscopy (Gupta et al., 2020). In case of plasma membranes, the diffusion coefficient is higher, and the domain confined diffusion of lipid probes is lower for the inner leaflet as compared to the outer leaflet. We showed that in the asymmetric LPS R45/PL aGUVs no domains were observable at temperatures above 25°C, but below 25°C domains in the outer and inner leaflet appeared in a registered phase (Figure 6A). Consequently, the phase transition temperature of around 25°C for asymmetric GUVs does not correspond to that of 42°C for mixed LPS R45/PL liposomes (SUVs). Therefore, we suggested that the inter-leaflet coupling between the LPS R45 leaflet and the PL leaflet is strong. In general, membrane domain registration and anti-registration describe the inter-leaflet domain dynamics, which are mainly determined by the inter-leaflet coupling (Zhang and Lin, 2019). In case of symmetric LPS R45/LPS R45 GUVs a clear registered domain formation could be observed (Figure 6B). Interestingly, in case of GUVs composed of LPS R45 on one leaflet and LPS R595 on the other leaflet a clear anti-registration could be observed (Figure 6D). Thus, both registration and anti-registration, can occur. Since, the natural LPS preparations are mixtures of different molecules with variations in number and length of the fatty acid chains and differences in the non-stoichiometric substitutions a simple interpretation of these data is not possible. Further experiments using various well-defined preparations will follow. Bossa et al. (2019) suggested that registered domains are not structurally but energetically connected across the bilayer. Furthermore, they define two distinct inter-leaflet coupling mechanisms: (i) a thermodynamically driven one due to the presence of a compositional mismatch between the two leaflets and (ii) a structurally driven one due to the presence of transmembrane proteins. WANG et al. observed in asymmetric vesicles that the inhibition of outer-leaflet ordered-domain formation by inner-leaflet lipids decreased. He explains this by an increased ability of outer-leaflet lipids to form an ordered state by themselves (Wang and London, 2018). From coarse-grained molecular dynamics simulations Fowler et al. (2016) proposed a two-step model in which membranes undergo a shift from an anti-registered phase to registered symmetric phase after bilayer equilibration. In summary, asymmetric LPS-containing bilayers can have registered and/or anti-registered phases of different sizes depending on temperature and lipid composition.

The asymmetry of membranes not only plays a decisive role in the thermodynamic behavior of pure lipid components but can also be decisive in the interaction with peptides and proteins. In many cases, proteins are responsible for the expression of asymmetry or its maintenance. Proteins, such as flippases, floppases, and scramblases influence the asymmetry in a targeted manner, but other proteins can also influence the distribution of lipids. On the other hand, asymmetry can have an influence on the binding, incorporation, orientation and functions of peptides and proteins. We used the polycationic antimicrobial peptide LL-32, which binds in a charge-dependent manner to lipid membranes. Therefore, the interaction with the highly negative charged LPS leaflet should be stronger than with the PL leaflet having a lower surface charge density (Hagge et al., 2006). Furthermore, it has been shown before that the permeabilization is strongly concentration-dependent (De Miguel et al., 2019). Both effects have been perfectly visible using the asymmetric LPS/PL GUVs. The micrographs in Figure 7 demonstrate the ability to measure the permeabilization of the GUVs by the influx of small quenching molecules such as KI. Only in case of a high enough LL-32 concentration pores or lesions were induced. Fluorescence spectroscopy allowed to integrate the effects over many aGUVs. The addition of LL-32 led to a fast increase of the fluorescence of the lipid-bound dye located in the inner leaflet by reducing the self-quenching caused by an insertion of the peptide into the hydrophobic region of the bilayer (Figure 8). After the initial increase, the fluorescence decreased because of an influx of the quenching molecule Na_2_S_2_O_4_ into the vesicle. In case of GUVs composed of LPS on the outer leaflet the permeabilization is more pronounced as compared to the one composed of PL on the outer leaflet. Bacterial porins intercalate into lipid membranes depending on the asymmetry of the bilayer. It has been shown for PhoE that the insertion only occurs from the PL side but not from the LPS side of planar lipid bilayers (Hagge et al., 2002). OmpF shows the same behavior using Montal-Mueller membranes (to be published elsewhere). The same effect can be seen in the quenching experiments using the asymmetric GUVs composed of LPS or PL on the outer leaflet. Only in case of PL facing the outer leaflet OmpF addition led to protein insertion into the bilayers inducing influx of the quencher (Figure 8).

In summary, asymmetric GUVs are an excellent tool to characterize specific membrane properties and their influence on the interaction with peptides and proteins. It has been shown that asymmetric membranes are more than just the sum of their two halves and that the function of proteins is strongly influenced by the specific lipid asymmetry. Therefore, asymmetric GUVs mimic the outer membrane of Gram-negative bacteria more closely and comprise a unique tool to test and study the lipid-mediated resistance of bacteria.

## Data Availability Statement

All datasets presented in this study are included in the article/Supplementary Material.

## Author Contributions

LP and AD contributed equally to the manuscript. AD, MK, LP, and TG designed the experiments. AB performed the AFM experiments. SH performed the Montal-Mueller experiments. AD, SG, and LP prepared asymmetric vesicles. MK and LP conducted the fluorescence microscopy. SG performed the lipid flip-flop and fluorescence spectroscopic experiments. MW contributed reagents and materials. AD prepared the first draft of the manuscript. LP, TG, and CN wrote the manuscript. All authors contributed to the interpretation of primary data and the discussion.

## Conflict of Interest

The authors declare that the research was conducted in the absence of any commercial or financial relationships that could be construed as a potential conflict of interest.
