# Supplementary material for: The Beauty of Asymmetric Membranes: Reconstitution of the Outer Membrane of Gram-Negative Bacteria
Source: Front Cell Dev Biol. 2020 Jul 14;8:586. doi: 10.3389/fcell.2020.00586 (PMC7381204; doi:10.3389/fcell.2020.00586)
Supplement: Supplementary file 1 [file Image_1.pdf]

Fig. S1

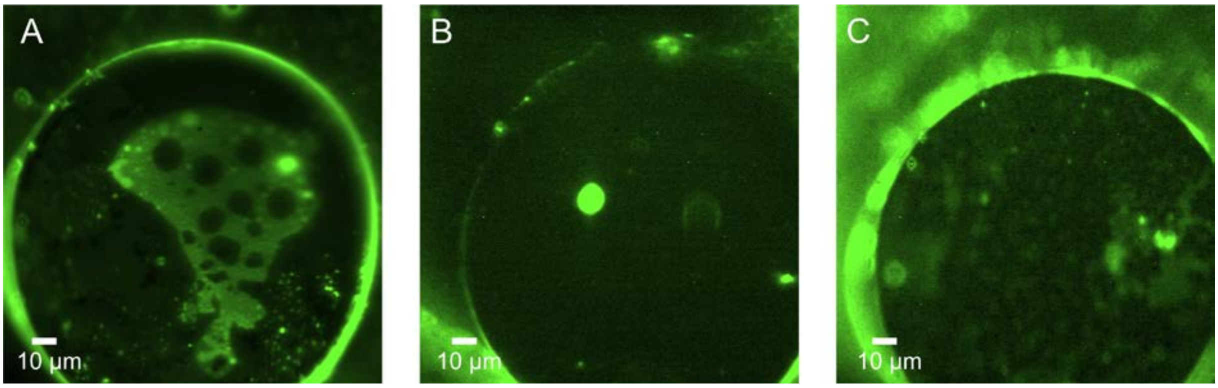

Fluorescence microscopic image of a planar monolayer (first step in the Montal-Mueller setup) prepared with 1 mol% NBD-PE doped LPS R45 monolayer over the aperture of a septum at 37°C (A), after heating to 42°C (B) and subsequent cooling to 37°C (C) (buffer composition: 100 mM KCl, 5 mM MgCl2, 5 mM HEPES, pH 7). The exposure times for these images were 200 ms (A and C) and 1 s (B).
